# Extracellular Matrix Modulation Is Driven by Experience-Dependent Plasticity During Stroke Recovery

**DOI:** 10.1007/s12035-017-0461-2

**Published:** 2017-03-13

**Authors:** Miriana Jlenia Quattromani, Mathilde Pruvost, Carla Guerreiro, Fredrik Backlund, Elisabet Englund, Anders Aspberg, Tomasz Jaworski, Jakob Hakon, Karsten Ruscher, Leszek Kaczmarek, Denis Vivien, Tadeusz Wieloch

**Affiliations:** 1 Laboratory for Experimental Brain Research, Division of Neurosurgery, Department of Clinical Sciences, Lund University, BMC A13, 22184 Lund, Sweden; 2 INSERM UMR-S U919, Serine Proteases and Pathophysiology of the Neurovascular Unit, Université Caen Basse Normandie, GIP Cyceron, F-14074 Caen, France; 3 Division of Oncology and Pathology, Lund University Hospital, 22185 Lund, Sweden; 4 Rheumatology and Molecular Skeletal Biology, Department of Clinical Sciences, Lund University, BMC C12, 22184 Lund, Sweden; 5 Laboratory of Neurobiology, Nencki Institute of Experimental Biology, 02-093 Warsaw, Poland; 6 Department of Clinical Research, Caen University Hospital, CHU Caen, 14000 Caen, France

**Keywords:** Stroke recovery, Experience-dependent plasticity, Extracellular matrix, Perineuronal nets, Somatosensory cortex, Proteases

## Abstract

Following stroke, complete cellular death in the ischemic brain area may ensue, with remaining brain areas undergoing tissue remodelling to various degrees. Experience-dependent brain plasticity exerted through an enriched environment (EE) promotes remodelling after central nervous system injury, such as stroke. Post-stroke tissue reorganization is modulated by growth inhibitory molecules differentially expressed within the ischemic hemisphere, like chondroitin sulfate proteoglycans found in perineuronal nets (PNNs). PNNs in the neocortex predominantly enwrap parvalbumin-containing GABAergic (PV/GABA) neurons, important in sensori-information processing. Here, we investigate how extracellular matrix (ECM) proteases and their inhibitors may participate in the regulation of PNN integrity during stroke recovery. Rats were subjected to photothrombotic stroke in the motor cortex, and functional deficits were assessed at 7 days of recovery. Sham and stroked rats were housed in either standard or EE conditions for 5 days, and infarct volumes were calculated. PNNs were visualized by immunohistochemistry and counted in the somatosensory cortex of both hemispheres. mRNA expression levels of ECM proteases and protease inhibitors were assessed by RT-qPCR and their activity analyzed by gel zymography. PNNs and protease activity were also studied in brains from stroke patients where similar results were observed. EE starting 2 days after stroke and continuing for 5 days stimulated behavioral recovery of limb-placement ability without affecting infarct size. EE promoted a decrease of PNNs around PV/GABA neurons and a concomitant modulation of the proteolytic activity and mRNA expression of ECM proteases and protease inhibitors in the somatosensory cortex. This study provides molecular targets for novel therapies that could support rehabilitation of stroke patients.

**Electronic supplementary material:**

The online version of this article (doi:10.1007/s12035-017-0461-2) contains supplementary material, which is available to authorized users.

## Introduction

According to the World Health Organization, approximately 15 million people suffer stroke worldwide each year. Of these, five million die and another five million are permanently disabled, making stroke a global leading cause of death and long-term disability [1, 2]. Stroke treatment options are restricted to the few hours after the accident [3, 4], and beyond that therapeutic window, treatment is limited to supportive care and rehabilitation. Yet, most stroke patients recover to some extent, demonstrating that remarkable brain plasticity is retained after the injury and that the recovery process should emerge as a promising target in stroke research [5].

The current experimental evidence strongly suggests that multisensory stimulation by changes in the environment (such as enriched environment (EE)) as well as task-driven experience confer an additive benefit on behavioral recovery after brain insults such as stroke, most likely because of their impact on brain plasticity [6, 7]. This promising approach has been recently translated into the clinical practice [8–10].

EE provides laboratory animals with novelty and extra space, allowing different forms of multisensory stimulation ranging from social grouping to enhanced motor activity. EE results in improved cognitive and sensorimotor functions both in naive rodents and in animals with brain lesions such as those occurring after a stroke. In animals which have suffered a stroke, robust behavioral effects are seen during recovery and are probably related not only to neuronal plasticity in the peri-infarct cortex but also in remote brain areas [7, 11–13].

After stroke, a number of growth inhibitory molecules are differentially expressed within the peri-infarct region, leading to inhibition of neuronal plasticity [14, 15]. Interestingly, in areas more distant from the infarct, there is a reduction in the number of these inhibitory molecules, particularly in chondroitin sulfate proteoglycans (CSPGs) and their formation into dense lattice-like structures termed perineuronal nets (PNNs) [15–18]. PNNs are considered a specialized form of extracellular matrix (ECM) with a major role in establishing and stabilizing connectivity in the brain by restricting plasticity. They are typically localized around inhibitory neurons [19], but they have been also described around excitatory pyramidal neurons [20–22]. CSPGs present in PNNs are lecticans and include aggrecan, brevican, neurocan, and versican, with aggrecan exclusively present in PNNs and therefore being a specific marker for PNNs in the adult brain [23]. We have recently shown that 9 weeks of EE after stroke induce a further reduction in the number of these inhibitory molecules, especially Cat-315^+^ (aggrecan-containing) PNNs [12], supporting the idea that part of the peri-infarct and remote cortex possesses an environment for post-stroke response strategies [24]. Several proteases are involved in the remodelling of ECM and PNNs, including tissue plasminogen activator (tPA) [25], type 4 disintegrin and metalloproteinase with thrombospondin motifs (ADAMTS-4) [25], and matrix metalloproteinases (MMPs) such as MMP-2 and MMP-9 [26–28]. tPA is a serine protease present in the blood, in the brain and at the interface between the blood and the brain [29]. In the brain parenchyma, tPA is expressed and released by endothelial cells, neurons, astrocytes, microglia, and oligodendrocytes. tPA contributes to ECM degradation, influence cell migration, neuronal plasticity, death and survival of neurons, endothelial cells, and oligodendrocytes [29]. ADAMTS-4 has been described in the brain parenchyma, mainly in astrocytes, neurons, microglia, and monocyte/macrophages and is involved in several central nervous system (CNS) functions, including neurorepair, remyelination, angiogenesis, and inflammation [30]. Cleavage by tPA produces the active form of ADAMTS-4, which then degrades inhibitory CSPGs, leading to increased neurite growth and subsequent functional recovery [25]. The MMP family of proteases act outside the cells and are therefore associated with ECM remodelling. They are expressed in neurons, glia, and endothelial cells and are involved in a number of physiological and pathological conditions, including development, tissue remodelling, inflammation, and metastasis [31]. Current data suggest that MMPs (such as MMP-2 and MMP-9) mediate beneficial dendritic plasticity and ECM remodeling at delayed stages after stroke [28]. Multiple studies showed increased expression and activity of MMP-2 and MMP-9 after brain injury (such as stroke) during the recovery phase [28, 31]. In particular, MMP-9 has been implicated in synaptic plasticity, learning and memory, and tissue remodelling in response to enhanced neuronal activity [32, 33]. Inhibitors of these proteases (such as neuroserpin, tissue inhibitor of metalloproteinases TIMP-1 and TIMP-3) are also considered strategic in promoting neuronal remodelling and neuroplasticity in the CNS [29, 34].

The aim of this study was to further investigate the influence of multisensory brain stimulation provided by EE after stroke on PNN integrity and on changes in expression/activity of MMP-2 and MMP-9, tPA, ADAMTS-4, and their inhibitors.

## Materials and Methods

### Rats

A total of 40 adult male Sprague-Dawley rats (8–11 weeks, Charles River) were used in this study and housed under reverse light conditions with free access to food and water. Physiological data are given in Supplement Table 1.

### Human Postmortem Brain Tissue

A total of ten stroke (*n* = 5) and non-stroke (*n* = 5) cases were used in this study. Age, sex, cause of death, and postmortem delay are given in Supplement Table 2.

### Experimental Stroke

Animals underwent permanent focal ischemia by photothrombosis (PT) in the left hemisphere as previously described [12, 35] or sham operation (same procedure as PT, but without illumination). During surgery, the body temperature of the animals was kept at 37 °C using a self-regulating heating pad. Briefly, isoflurane-anesthetized rats (2% in O_2_ under spontaneous ventilation) received intravenous injections of the photosensitive dye Rose Bengal (10 mg/ml, Sigma, USA) in the tail vein. The skin above the skull was incised, and the brain was illuminated through the exposed skull with cold light (KL 1500 LCD, Schott) for 15 min at a stereotactically defined position (0.5 mm laterally and +4/−4 mm anterior/posterior to bregma), producing an approximate irradiation area of 8 × 4 mm^2^. Incisions were sutured, and animals were allowed to awake from anesthesia while on a heating pad and returned to their home cages.

### Behavioral Assessment and Randomization

#### Paw Placement Test

Sensorimotor function and touch sensation were assessed by the paw placement test, which provides information on the tactile/proprioceptive response to limb stimulation [12, 36]. Testing at all times was performed by the same investigator. In brief, every rat was placed with all paws on a bench surface and hand-held in a horizontal position. Facial contact stimuli were avoided by supporting the rat’s chin and holding its head 45° upward. The paws to be tested were gently pushed along the edge in order to loose contact with the bench surface. The ability of the rat to place the limb back on the bench surface when the animal was moved along its edge was evaluated with the following score: (1) prompt placement of the limb on the table, (0.5) incomplete placing of the limb, (0) no response, with persistent extension of the limb and paw.

One day before PT or sham operations, all animals had a score = 1 in each of the four paws, indicating that no deficit in sensorimotor function was present before the surgeries.

#### Differential Housing Conditions

Two days after surgeries, both PT and sham animals were assigned to either group I or II (*n* = 20 each) and randomized into (standard) STD or EE cages [12] where they were housed for 5 days. Rats housed in STD conditions had one cage mate, and rats in enriched cages had up to five mates. Multilevel EE cages were equipped with various colored objects such as plastic tunnels, removable platforms, grids, pipes, ropes, ladders, and chains. The disposition of the objects was changed twice/week.

**Fig. 1:**
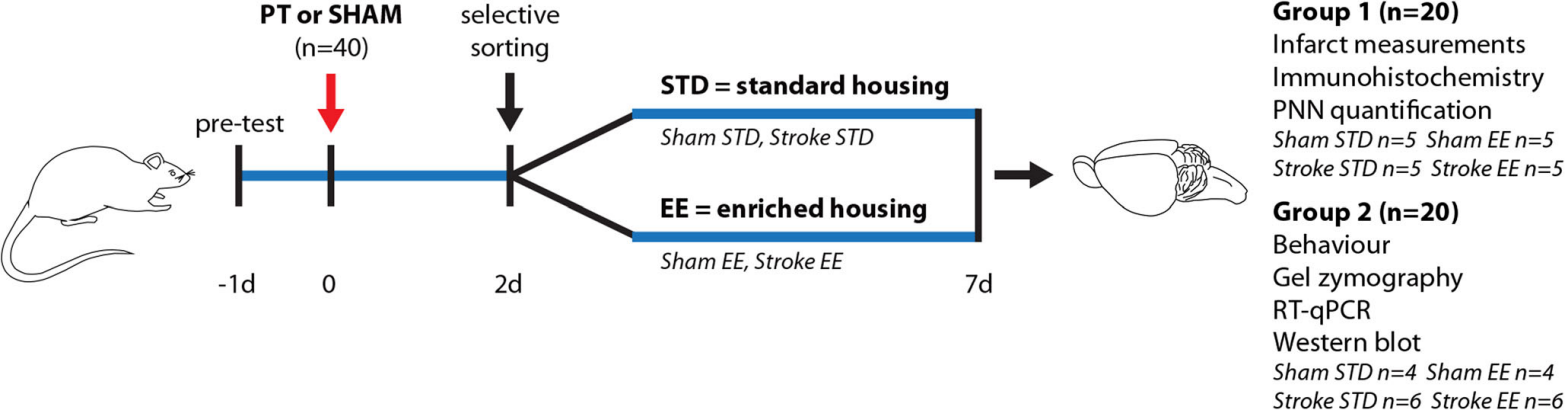
Experimental design. Rats were pre-tested to assess their full limb placement ability 1 day before photothrombosis (PT) or sham operations. Functional deficits were assessed 2 days after PT and rats were selectively sorted into differential housing conditions (standard, *STD*; enriched, *EE*). Differential housing was continued for the five following days. At 7 days of recovery, brains were perfusion-fixed (group I) or frozen (group II) for further analyses

The animals were divided as described in Fig. 1. Brains from groups I and II were collected and handled accordingly to postmortem analyses. Brain samples from group I were used for immunohistochemical analyses (infarct measurements and cell counting). Animals from group II were used for behavioral assessments on day 7, and after sacrifice, the brains from this group were used for molecular biology experiments (gel-zymography, western blot, RT-qPCR).

### Brain Preparation from Rats

#### Group I

Rats were anesthetized with 4% isoflurane and transcardially perfused with 0.9% NaCl followed by cold phosphate-buffered 4% paraformaldehyde (PFA, Sigma, USA), pH 7.4. Brains were postfixed overnight in PFA and equilibrated in phosphate-buffered 30% sucrose for 48 h at 4 °C. Brains were sectioned in the coronal plane on a microtome at 30-μm intervals. Sections were collected and stored at −20 °C in an antifreeze solution made in phosphate buffer containing 30% glycerol and 30% ethylene glycol.

#### Group II

Rats were anesthetized with 4% isoflurane and perfused with cold heparinized 0.9% NaCl. Brains were extracted and immediately frozen by immersion in isopentane and dry ice. Brains were then stored at −80 °C until dissection. On the day of the dissection, frozen brains were moved to a −20 °C chamber and placed in a rat brain matrix. Two brain slices 2-mm-thick (2.20 to −3.30 mm from bregma) were excised using a scalpel blade. The primary somatosensory cortex on both ipsilateral and contralateral hemispheres was cut out according to Paxinos and Watson [37]. In the ipsilateral hemisphere, the sampling started 1 mm after the end of the lesion. Samples were transferred to tubes and stored at −80 °C until further use.

### Brain Preparation from Patients

Following autopsy, cortical fresh tissue from the infarct core, peri-infarct, and remote regions were dissected out by a pathologist, immediately frozen and stored at −80 °C. For further dissection, samples were temporarily moved to a −20 °C chamber where smaller specimens were excised and stored at −80 °C until protein extraction.

### Immunohistochemistry and Immunofluorescence (IHC/IF)

#### Rat Brain Tissue

Free-floating brain slices from group I were rinsed in phosphate-buffered solution (PBS), quenched in 3% H_2_O_2_ and 10% methanol for 15 min (IHC) and blocked in blocking solution (5% normal donkey serum, Jackson ImmunoResearch, UK, and 0.25% Triton X-100 in PBS) for 1 h at room temperature (RT). Sections were incubated overnight at 4 °C with primary antibodies (monoclonal mouse anti-CSPGs, 1:3000 for IHC and 1:1000 for IF, Cat-315/MAB1581, Millipore, USA; goat anti-parvalbumin (PV), 1:2500 for IF, PVG-214, Swant, Switzerland) diluted in blocking solution. Following rinses with 2% normal donkey serum and 0.25% Triton X-100 in PBS, the sections were incubated with appropriate secondary antibodies (donkey anti-mouse for IHC, Vector Laboratories, USA; donkey anti-mouse/goat conjugated with either Cy3 or DyLight 488 fluorescent dyes for IF, Jackson ImmunoResearch, UK) at a dilution of 1:400 in blocking solution for 90 min at RT. IHC visualization was achieved via the Vectastain ABC kit (Vector) using 3,3′-diaminobenzidine-tetrahydrochloride-dihydrate (DabSafe, Saveen Werner, Sweden), 8% NiCl, and 3% H_2_O_2_. Bright-field pictures were acquired using an Olympus BX60 microscope (Sweden). Fluorescent labelling was imaged using a confocal laser-scanning microscope (Zeiss LSM 510, Germany).

#### Human Brain Tissue

Five-micron-thick formalin-fixed paraffin-embedded slices were evaluated by a pathologist before serial sections were stained. The presence of infarct and infarct age was confirmed by microscopy of the tissue in the immediate proximity to the areas sampled and analyzed. Brain slices were deparaffinized, immersed in lectin buffer (LB: 0.1 M Tris-buffer pH 7.6, 1.45 M NaCl, 0.01 M MgCl_2_, 0.01 M CaCl_2_) for 1 h and quenched in 1% H_2_O_2_ and 90% methanol for 20 min. Following rinses in LB, antigen retrieval was performed by microwave boiling in 0.01 M citrate buffer pH 6.0 for 15 min and slices were left in the solution until they reached RT. After rinsing in LB, sections were blocked in 1% bovine serum albumin (BSA, Sigma, USA) for 1 h and incubated with biotinylated *Wisteria floribunda* agglutinin (WFA, 1:200, L1516, Sigma, USA) for 40 min at 37 °C in LB. After rinsing in LB, ABC and DAB steps followed the same protocol used for rat sections. Bright-field pictures were acquired using an Olympus BX60 microscope (Sweden).

### Infarct Volume Measurements

For each animal in group I, ten coronal 30-μm-thick brain sections with a distance of 1.0 mm were immunostained with a monoclonal mouse anti-NeuN antibody (MAB377, Millipore, USA) at a dilution of 1:1500. The sampling ranged from 4.20 to −3.80 mm from bregma and covered the entire rostro-caudal extension of the lesion. The non-lesioned area of the infarcted hemisphere and the non-lesioned contralateral hemisphere were outlined on each brain section using the ImageJ software (National Institute of Health, USA). Infarct volumes were determined by subtracting the area of the non-lesioned ipsilateral hemisphere from that of the intact contralateral hemisphere and calculated by volumetric integration for each animal [38].

### Cell Counting

#### Bright Field Microscopy

In group I, three coronal sections per brain (2.20, 0.48, and −3.30 mm relative to bregma) were stained for Cat-315 as described above. Composite micrographs of the whole ipsilateral and contralateral hemispheres were acquired through a ×4 magnification objective using the CellSens Dimension Software (Olympus BX60, Sweden). An optical grid was used to define distances and draw the boundaries of the primary somatosensory cortex in both hemispheres according to the Paxinos atlas [37]. The sizes of the areas of interest were 6.55 mm^2^ at 2.20 mm from bregma, 10.90 mm^2^ at 0.48 mm from bregma, and 8.34 mm^2^ at −3.30 mm from bregma.

#### Confocal Microscopy

One coronal section per brain (0.48 mm relative to bregma) was double-stained for Cat-315 and parvalbumin as described above. Three areas of interest within the somatosensory cortex (layers II–III) were acquired using a confocal laser-scanning microscope ×20 objective (Zeiss LSM 510, Germany). For analysis, the ImageJ software was used to discriminate between the different fluorophores. Cat-315^+^ cells, parvalbumin^+^ cells, and cells positive for both antibodies were counted. Data are presented as an average of the three regions of interest (ROIs).

### Protein Extraction and Quantification

For group II and human tissue, regions of interest were dissociated in TNT buffer, pH 7.4 (50 mM Trizma base, 150 mM NaCl, 0.5% Triton X-100) and centrifuged at 12,000*g* for 15 min at 4 °C. Supernatants were collected and protein determination performed with the Pierce BCA protein Assay Kit according to the manufacturer (ThermoFisher Scientific, USA). Twenty-microgram aliquots were prepared and stored at −80 °C until further use.

### Gel-Zymography Assays

#### MMP-2 and MMP-9 Zymography

Equal amounts of proteins (20 μg) were mixed with 5× sample buffer and separated at 4 °C under non-reducing conditions on 8% SDS-PAGE gels copolymerized with FITC-labeled gelatin (FITC 200 μg/ml, Sigma, USA; gelatin 20 mg/ml, Sigma, USA) as in [39]. Gels were washed in 2.5% Triton X-100 (2 × 30 min, RT) to remove SDS, and then incubated in activation buffer (50 mM Tris-HCl pH 7.5, 10 mM CaCl_2_, 1 μM ZnCl_2_, 1% Triton X-100, 0.02% NaN_3_) for 48 h at 37 °C to allow in-gel MMP renaturation [40]. Zymograms were digitized with a ChemiDoc XRS+ system (Bio-Rad, USA) under UV light and proteinase activity quantified by densitometry (ImageJ, USA).

#### tPA Zymography

Equal amounts of proteins (20 μg) were mixed with 5× sample buffer and separated at 4 °C under non-reducing conditions on 12% SDS-PAGE gels copolymerized with casein (10 mg/ml, Sigma, USA) and plasminogen (2.5 μg/ml, Calbiochem, USA). Gels were washed at 4 °C with cold 2.5% Triton X-100 (2 × 30 min) and cold distillated water (3 × 10 min), and then incubated in activation buffer (100 mM glycine pH 8.3, 10 mM EDTA) for 5.5 h at 37 °C to allow caseinolysis. Gels were stained with Coomassie Blue R-250 and destained with 40% methanol and 10% acetic acid. Zymograms were digitized with a CanoScan 8800F (Canon, USA) and proteinase activity quantified by densitometry (ImageJ, USA).

### Western Blot

#### β-Dystroglycan (β-DG)

Equal amounts of proteins (20 μg) were mixed with 5× sample buffer and separated at RT under reducing conditions on 10% SDS-PAGE gels. Proteins were transferred onto polyvinylidene difluoride (PVDF) membranes (Millipore, USA) for 1 h at RT and blocked with a solution containing 5% non-fat dry milk for 1 h at RT. Membranes were incubated overnight at 4 °C with primary antibody monoclonal mouse anti β-DG (1:500, ab49515, Abcam, UK) in 5% BSA. On the next day, membranes were incubated with a horseradish peroxidase (HRP)-conjugated anti-mouse IgG (1:10,000, A3862, Sigma, USA) for 1 h at RT. Protein bands were visualized by exposing the membranes to a CCD camera (LAS1000, Fujifilm, USA) using a chemiluminescent HRP substrate kit (Millipore, USA). Membranes were stripped and re-probed with an anti-β-actin HRP-conjugate (1:75,000, A3854, Sigma, USA). After densitometric analysis using the ImageJ software (National Institute of Health, USA), primary antibody levels were normalized to β-actin expression.

#### Aggrecan

Protein samples (20 μg) were treated with digestion buffer containing 100 mM Tris-HCl pH 8.0, 30 mM NaAc, 5 mM EDTA and protease inhibitors (Complete EDTA-free, Roche, Germany) with (+) or without (−) chondroitinase ABC (ChABC) (0.5 U/ml, Sigma, USA) at 37 °C for 3 h to remove chondroitin sulfate chains from the aggrecan proteoglycan. ChABC treatment induces a gel shift of the protein targets for the Cat-315 antibody, which we used to detect aggrecan. The shift diminishes the smear of immunoreactive aggrecan peptides, leaving a marked protein band at >250 kDa and weaker bands at lower molecular weights.

Proteins were then denatured and processed as above. Western blots for the Cat-315 antibody (1:5000, MAB1581, Millipore, USA) were ran on 4–15% gradient SDS-PAGE gels.

### RNA Extraction and Quantification

In group II, total RNA was isolated from our regions of interests with TRI reagent (Sigma, USA) according to the manufacturer. Total RNA was treated with TURBO DNase (Ambion, France) to avoid DNA contamination and was quantified by spectrophotometry (NanoDrop Technologies, USA). First-strand cDNA synthesis was performed from 1 μg of total RNA with the M-MLV Reverse Transcriptase (Invitrogen, France) in a total volume of 20 μL with the following cycle conditions: 37 °C (50 min); 70 °C (15 min). RT-qPCR was performed from 1 μL of 1:20 diluted cDNA in 15 μL total of a 1× solution of iQ SYBR Green Supermix (Bio-Rad, France) containing 200 nM of each primer. Based on mRNA coding sequences (www.ensembl.org), rat-specific primers (see Table 1) were designed by using the Primer3Plus software (http://www.bioinformatics.nl/cgi-bin/primer3plus/primer3plus.cgi). Assays for the 40 samples were run in triplicate on the CFX96 real-time system c1000 thermal cycler (Bio-Rad, France), with the following cycle conditions: 95 °C (3 min); [95 °C (2 s), 60 °C (20 s)] × 39; 70 °C (30 s). To investigate the best normalization process for reference genes, we used the geNorm algorithm associated to qBase+™ software and the Normfinder algorithm (Supplement Fig. 1) as previously described [41].

**Table 1:** Reference genes and primer sequences

Name	Forward	Reverse	Accession number	Transcripts length (base pairs)	Primer efficiency (%)
Housekeeping gene study					
*Actb*	agccatgtacgtagccatcc	accctcatagatgggcacag	NM_031144.3	115	100.8
*Gapdh*	gtgatgctggtgctgagta	gcggaaggggcggagatg	NM_017008.4	115	77.2
*Hmbs*	aggatgggcaactgtacctg	aactgtgggtcatcctctgg	NM_013168.2	130	85.1
*Hprt1*	gcagactttgctttccttgg	agaggtccttttcaccagca	NM_012583.2	79	86.3
*Ppia*	catcctgaagcatacaggtc	catccagccactcagtctt	NM_017101.1	107	98.7
*Ppib*	caagacctcctggctagacg	ttctccaccttccgtaccac	NM_022536.2	81	87.6
*Rpl13a*	gatgaacaccaacccgtctc	atcccatccaacaccttgag	NM_173340.2	141	90.5
*Sdha*	cgagatccgtgaaggaagag	ctctgagatcccaggcagac	NM_130428.1	109	90.0
Gene expression study					
*Ppib* ^a^	caagacctcctggctagacg	ttctccaccttccgtaccac	NM_022536.2	81	98.9
*Rpl13a* ^a^	gatgaacaccaacccgtctc	atcccatccaacaccttgag	NM_173340.2	141	98.9
*Adamts4*	aactccctgttccccagact	tagcttcagggccaggtaga	NM_023959.1	271	91.4
*Mmp9*	cactgtaactgggggcaact	cacttcttgtcagcgtcgaa	NM_031055.1	150	103.8
*Plat*	tctgccgcccactgctttg	cgaatgtctgctcctcctctcc	NM_013151.2	103	106.7
*Serpin1*	atgaggctggtggcatctac	gatcagctgtggtttgagca	NM_053779.1	131	99.8
*Timp1*	ggttccctggcataatctga	atggctgaacagggaaacac	NM_053819.1	99	101.2
*Timp3*	gctgtgcaactttgtggaga	aattgcaacccaggtggtag	NM_012886.2	86	93.6

^a^Used as housekeeping genes

A housekeeping gene study preceded the real gene expression analysis. By using the geNorm and the Normfinder methods, we studied the stability of eight common reference genes under our experimental conditions (β-actin “*Actb*,” glyceraldehyde-3-phosphate dehydrogenase “*Gapdh*,” hydroxymethylbilane synthase “*Hmbs*,” hypoxanthine-guanine phosphoribosyltransferase “*Hprt*,” peptidylprolyl isomerase A or cyclophilin A “*Ppia*,” peptidylprolyl isomerase B or cyclophilin B “*Ppib*,” ribosomal protein L13a “*Rpl13a*,” succinate dehydrogenase complex subunit A “*Sdha*”). Finally, two reference genes (“*Ppib*” and “*Rpl13a*”) were chosen for normalization in the study of our genes of interest (a disintegrin and metalloproteinase with thrombospondin motifs 4 “*Adamts4*,” metalloproteinase 9 “*Mmp9*,” tissue plasminogen activator “*Plat*,” neuroserpin “*Serpin1*,” tissue inhibitor of metalloproteinase 1 “*Timp1*,” tissue inhibitor of metalloproteinase 3 “*Timp3*”).

### Statistical Analysis

Statistical analysis was performed using the PRISM 7 software (GraphPad, USA). In all experiments, *P* < 0.05 was considered significant. Data are presented as means ± standard error of the mean (SEM) unless otherwise stated. When comparing two groups, the two-tailed unpaired Student’s *t* test was used in all cases except for behavioral analysis where the Mann-Whitney *U* test was employed. Ordinary one-way ANOVA was used when comparing more than two groups and in significant cases followed by the Bonferroni’s multiple comparisons test.

## Results

### EE Promotes Behavioral Recovery of Limb Placement Ability After PT Without Affecting Infarct Size

**Fig. 2:**
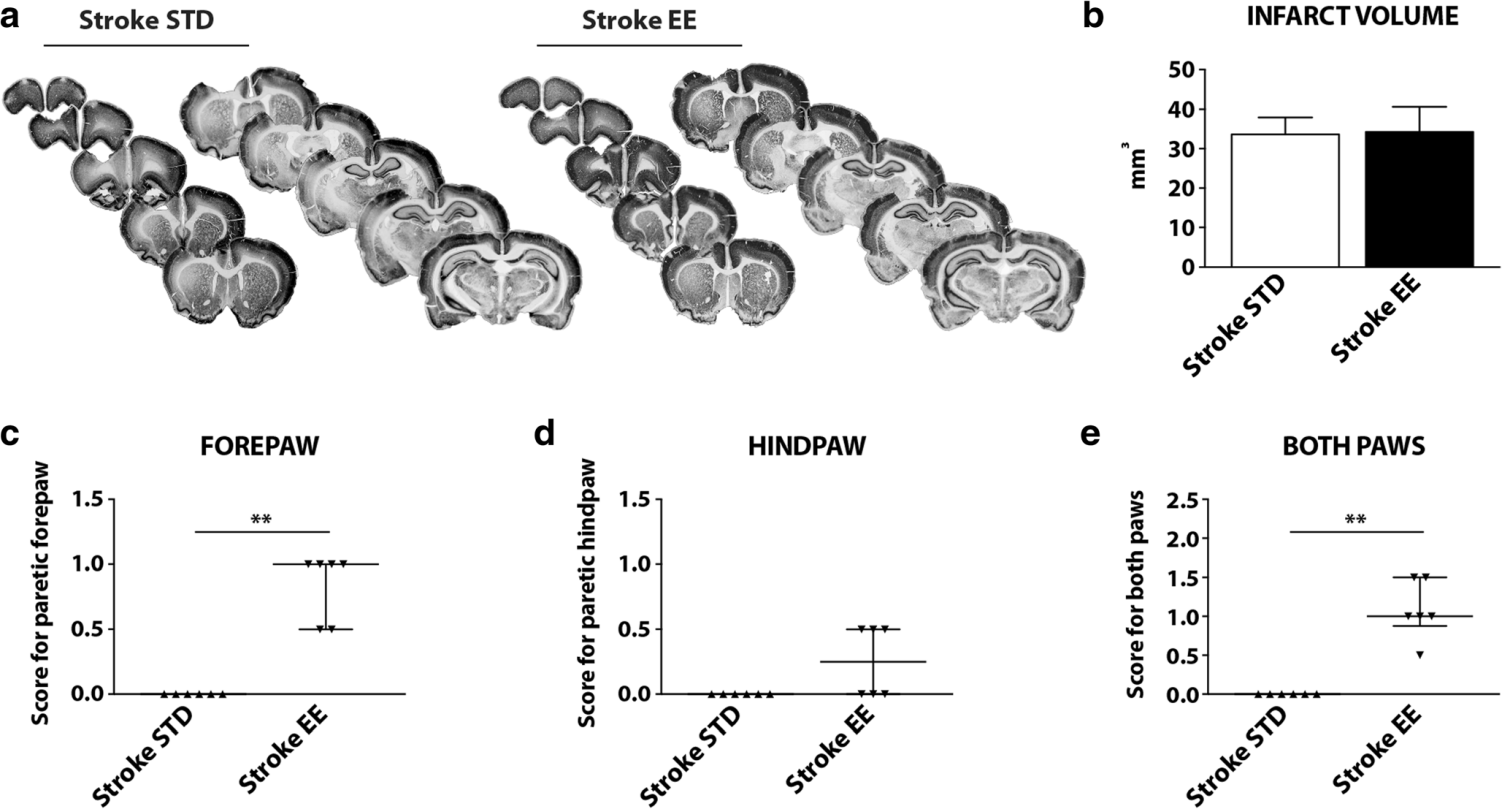
Infarct measurements and paw placement test. **a** Serial coronal slides of a representative brain infarct from the stroke standard (*STD*) and stroke enriched (*EE*) groups. **b** Infarct volume measurements 7 days after photothrombosis. Two-tailed unpaired Student’s *t* test, *n* = 5 for each group. **c**–**e** Limb placement ability of rats subjected to photothrombotic stroke (PT, left hemisphere). Scores are shown as individual data points with group median and interquartile range. During the pretest (before surgeries), all rats exhibited a score = 1 in all four paws, indicating no deficits. At day 2 of recovery, all rats subjected to PT (stroke STD; stroke EE) exhibited a score = 0 on the right forepaw and hindpaws, indicating full deficits in the placement of both limbs contralateral to the lesion. **c** Scores for forepaw placement (**P < 0.01), **d** hindpaw placement, and **e** sum of the scores for both paws (**P < 0.01). Mann-Whitney *U* test, *n* = 6 for each group

Consistent with previous work [12], the PT procedure resulted in local thrombosis of cortical microvessels and subsequent ischemic focal infarction in the left primary motor cortex. No signs of lesion could be detected in sham-operated animals (data not shown). Figure 2a shows a comparable localization of the lesion for the stroke STD and stroke EE groups. The lesion extended from the cortical surface down to the corpus callosum, without transecting the white matter. Importantly, the infarct volume at 7 days after PT and 5 days after differential housing conditions was 33.64 ± 4.28 mm^3^ for the stroke STD animals and 34.28 ± 6.38 mm^3^ for the stroke EE animals (Fig. 2b). Therefore, no significant difference between the two groups was present, consistent with other work in which EE was initiated at 2 days after PT [12, 13].

On day 2, after stroke and prior to differential housing conditions, all animals subjected to sham operation showed no deficit in the paw placement test (score = 1 for both paws; data not shown). On the contrary, all animals subjected to PT exhibited a clear deficit in both forepaw and hindpaw function (score = 0 in the paws contralateral to the side of the lesion; data not shown). On day 7 of recovery, despite equivalent infarct volumes, we observed a significant improvement in limb placement ability (contralateral to the side of the lesion) in the stroke EE group compared with the stroke STD group (Fig. 2c–e). Sham animals did not show any deficit, and their scores are not included in the graphs. Animals housed for 5 days in EE conditions after PT exhibited a significant recovery of forepaw function (*P* < 0.01, Fig. 2c), with 66% of the animals showing complete recovery (score = 1, four rats out of six) and 33% showing improved function (score = 0.5, two rats out of six). Also, 50% of the stroke EE animals exhibit an improved hindpaw sensorimotor function (score = 0.5 in three rats out of six, Fig. 2d). Figure 2e shows the sum of scores for both paws, with a score = 2 indicating full recovery of the paws contralateral to the lesion (forepaw = 1 + hindpaw = 1). The entire stroke EE group developed some improved function 7 days after stroke, with one third of the rats scoring 1.5 points, half of them scoring 1 point and one quarter showing 0.5 point (*P* < 0.01). On the contrary, all animals housed in STD conditions showed complete deficit in both forepaw and hindpaw limb placement ability, described by the lowest score in the test (score = 0, six rats out of six, Fig. 2c–e).

Taken together, these data show that 5 days of EE conditions initiated 2 days after PT induce a robust recovery of forepaw function and improved recovery of hindpaw function.

### PT and EE Diminish the Number of PNNs and Increase the Level of Cat-315^+^ Peptides

**Fig. 3:**
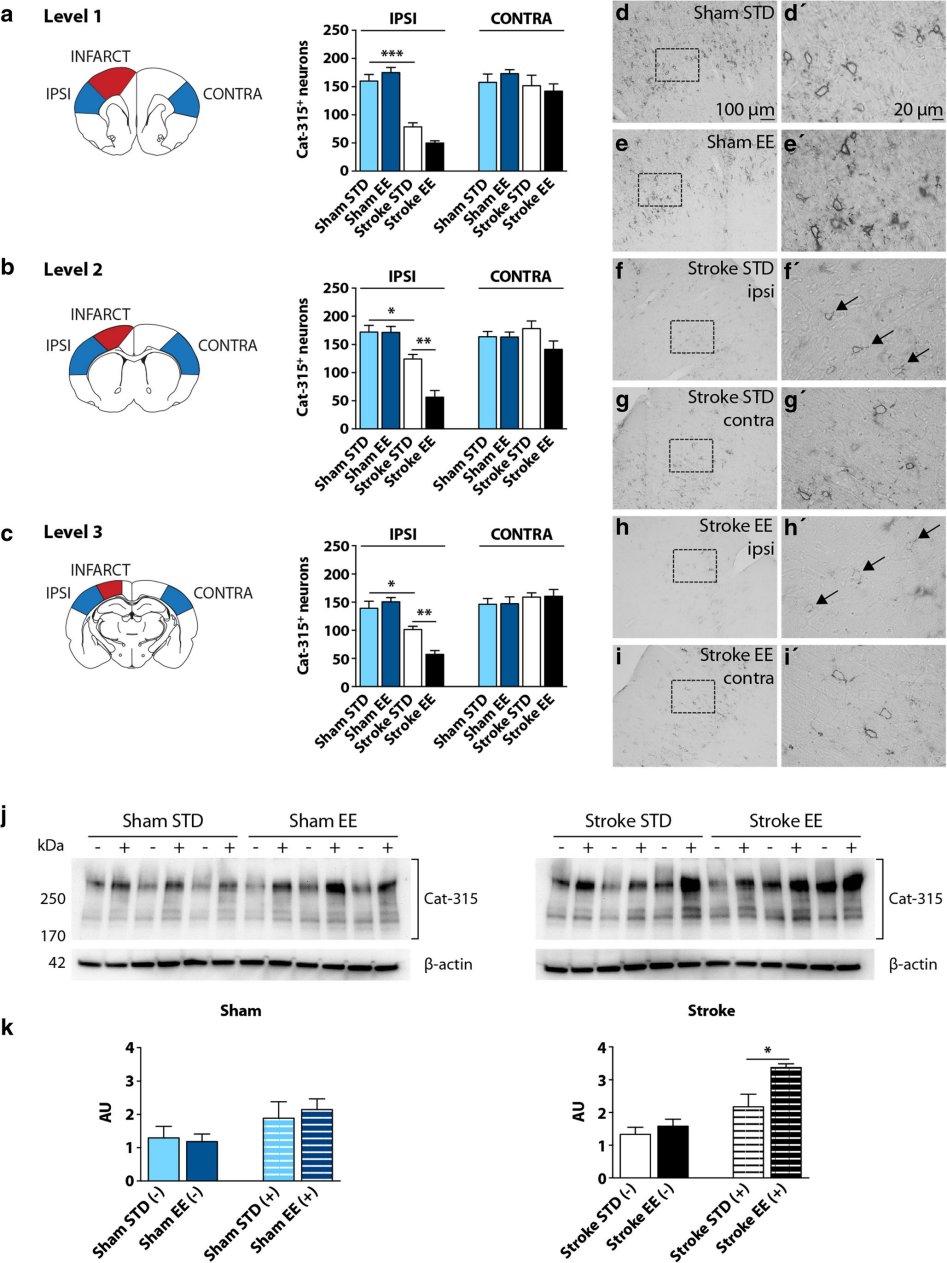
Bright-field cell counting, microscopy, and western blot of aggrecan in the rat somatosensory cortex. Cell counting of Cat-315^+^ PNNs at 7 days of recovery at different distances from bregma (**a** 2.20 mm, **b** 0.48 mm,**c** −3.30 mm). Results among groups were analyzed independently for the (IPSI) ipsilateral and (CONTRA) contralateral hemispheres. IPSI: **a** sham STD vs stroke STD (***P < 0.001); **b** sham STD vs stroke STD (*P < 0.05), stroke STD vs stroke EE (**P < 0.01); **c** sham STD vs stroke STD (*P < 0.05), stroke STD vs stroke EE (**P < 0.01). One-way ANOVA and Bonferroni’s multiple comparisons test, *n* = 5 for each group. **d-i'** Representative bright-field micrographs of Cat-315^+^ PNNs immunoreactivity in the somatosensory cortex.** d'-i'** Higher magnification. *Black arrows *are pointing at Cat-315^+^ PNNs degraded by stroke.** j** Representative Cat-315 western blots of protein homogenates from the somatosensory cortex of the IPSI hemisphere. Homogenates were digested with (+) or without (−) ChABC.** k** Densitometric analyses of Cat-315 western blots where data were normalized to β-actin expression. Significant differences were found in the comparison stroke STD (+) vs stroke EE (+), *P < 0.05. Two-tailed unpaired Student’s *t* test, *n* = 3 per group. *AU* arbitrary units, *STD* standard, *EE* enriched

To determine whether EE modulates the presence of aggrecan in the somatosensory cortex, cell counting was performed to study changes in its immunoreactivity at 7 days of recovery (Fig. 3).

Cell counting results are summarized in Fig. 3a (2.20 mm from bregma, level 1), Fig. 3b (0.48 mm, level 2), and Fig. 3c (−3.30 mm, level 3). These specific bregma distances always presented an infarct and were selected in order to cover the vast majority of the somatosensory cortex. Results among groups were analyzed independently for the ipsilateral and contralateral hemispheres. In both shams and stroke animals, PNNs were localized mainly between layers II and VI of the cortex and the immunoreactivity revealed a lattice-like structure surrounding the cell body and proximal dendrites. In sham animals, the distribution of PNNs varied substantially across cortical areas where the primary somatosensory cortex, auditory cortex, and retrosplenial cortex resulted richest in PNNs (data not shown). Statistical analysis showed significant differences among the four experimental groups in the ipsilateral hemisphere of all three levels (*P* < 0.001, Fig. 3a–c). A general decrease in the number of PNNs was seen after stroke and a further decrease in the number of nets was seen in the stroke EE group. In the contralateral hemisphere, these differences were not present among the groups and the number of PNNs was not affected by stroke. Importantly, differential housing conditions did not alter the number of PNNs in sham animals.

In the ipsilateral hemisphere of level 1 (Fig. 3a), post hoc comparisons showed significant difference between the sham STD and stroke STD groups (*P* < 0.001). Cell counting from level 1 show a reduction of 51 and 72% in the number of PNNs after stroke or stroke and EE conditions, respectively, compared to shams. In level 2 (Fig. 3b), the analysis showed differences similar to level 1, and here, the comparison between the stroke STD and the stroke EE groups reached statistical significance (sham STD vs stroke STD *P* < 0.05; stroke STD vs stroke EE *P* < 0.01). Cell counting from level 2 indicates a reduction in the number of PNNs of 28 and 67% in the stroke STD group and stroke EE group, respectively, compared to shams. Comparable results were seen in level 3 (Fig. 3c) (sham STD vs stroke STD *P* < 0.05; stroke STD vs stroke EE *P* < 0.01). In this level, the reduction seen in the number of PNNs was 27 and 62% when comparing shams with stroke STD and stroke EE, respectively.

Figure 3d–i′ shows representative micrographs of PNNs in the rat cortex. Figures 3d–e and d′–e′ (in higher magnification) illustrates the robust presence of nets in sham animals, thick and dispersed between layers II and IV. Figures 3f–g displays the nets of a stroke STD animal. In this case, the nets are quite reduced in number in the ipsilateral hemisphere compared to shams or the contralateral side. Also, the thickness of the nets seems altered by the stroke since they appear thinner (Fig. 3f′ and g′) compared to the shams animals. PNNs of a stroke EE rat are shown in Fig. 3h–i, where they appear barely visible in the ipsilateral hemisphere. Figure 3h′ displays the morphological appearance of some very thin and interrupted nets in the ipsilateral hemisphere of a stroke EE rat, which appear almost degraded compared to the contralateral side (Fig. 3i′).

To determine whether the EE-induced reduction of PNNs in the cortex after PT is accompanied by a change in the cortical levels of the aggrecan protein, western blot studies were performed on cortical homogenates 7 days after PT and 5 days after EE. Figure 3j shows representative western blots of protein samples from the somatosensory cortex of the ipsilateral hemisphere. Samples were treated with (+) or without (−) ChABC. The Cat-315 antibody detected a smear of immunoreactivity and multiple bands between ∼200 and 330 kDa on the western blots, which were quantified. Densitometric analyses of the sham groups did not show significant differences between the STD and EE groups (Fig. 3k). Likewise, among the stroke groups, protein levels were not altered in the absence of ChABC. Treatment with ChABC sharpened the protein bands and removed much of the smear above the main protein band at 250 kDa (Fig. 3j). In ChABC-treated samples, the integrated level of Cat-315^+^ peptides increased by 35% (*P* < 0.05) in the stroke EE group compared to the stroke STD group (Fig. 3k).

In summary, these data show that PT induces a reduction in the number of PNNs in the ipsilateral hemisphere of the somatosensory cortex visible 7 days after surgery. Interestingly, PT and 5 days of EE conditions further reduce the number of PNNs, which is visible in two of the three levels analyzed. The enhanced decrease of PNNs under EE conditions is accompanied by an increase in the levels of Cat-315^+^ aggrecan and what appears to be aggrecan fragments.

### EE Promotes a Decrease of Cat-315^+^ PNNs Around PV/GABA Neurons

**Fig. 4:**
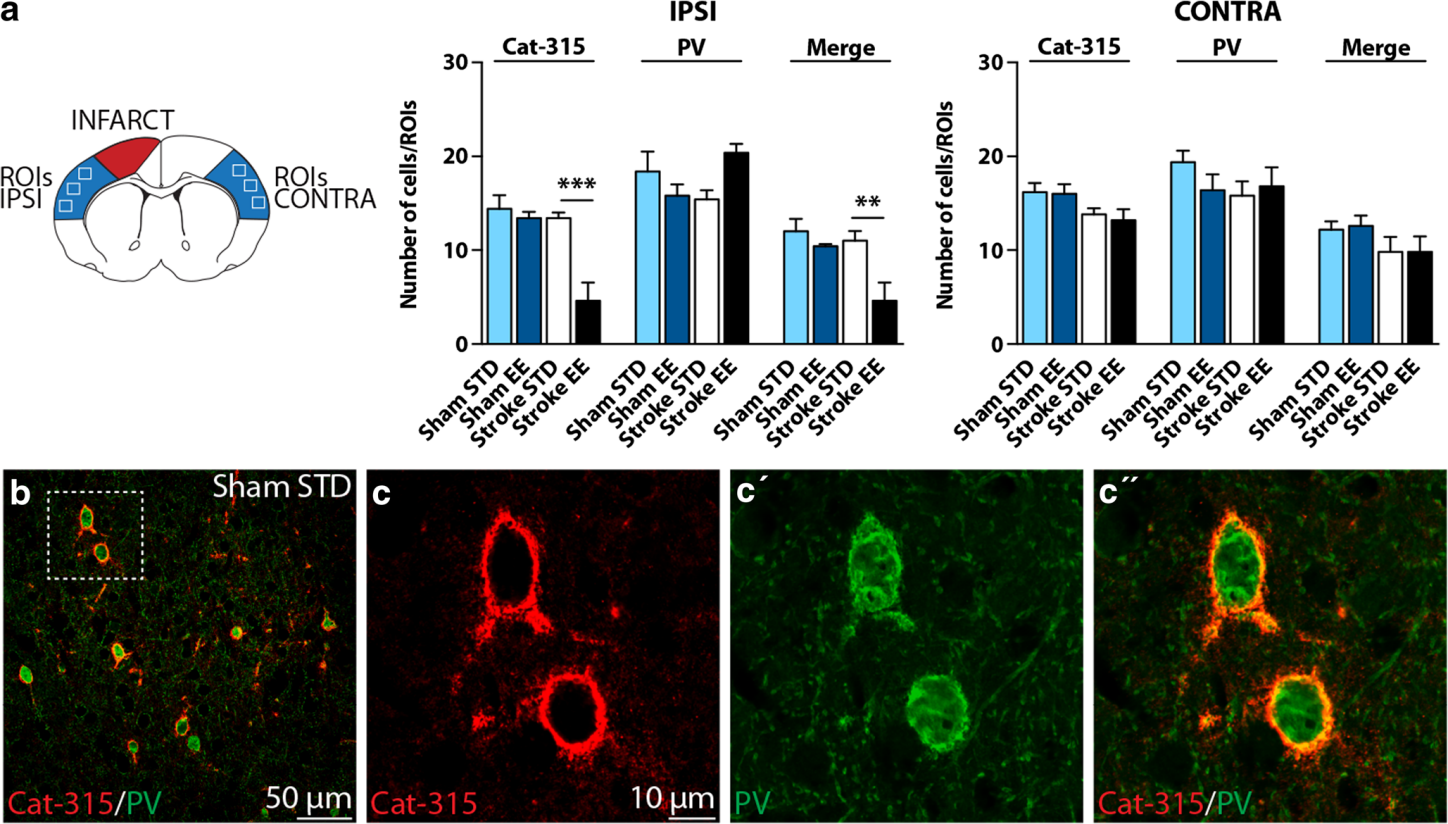
Confocal cell-counting and microscopy of parvalbumin (PV)^+^ and PV-aggrecan^+^ PNNs in the rat somatosensory cortex.** a** Results from the counting of nets stained with PV, Cat-315, and the nets that expressed both markers at 7 days of recovery (0.48 mm from bregma). Means are intended as mean average number of cells for three regions of interest (ROIs). Results among groups were analyzed independently for the Cat-315, PV, and merged stainings and for the ipsilateral hemisphere (IPSI) and contralateral hemisphere (CONTRA). IPSI: Cat-315 staining, stroke STD vs stroke EE (***P < 0.001); merged staining, stroke STD vs stroke EE (**P < 0.01).** b** Representative double immunostaining for PV (*green*) and Cat-315 (*red*) from the somatosensory cortex of a sham STD rat,** c-c''** higher magnification. One-way ANOVA and Bonferroni’s multiple comparisons test, *n* = 5 for each group. *STD* standard, *EE* enriched (color figure online)

To reveal the identity of neurons that were enwrapped with PNNs in the somatosensory cortex, we performed a double immunofluorescent staining of PNNs with Cat-315 (aggrecan) and PV (marker for a subset of GABAergic neurons). In Fig. 4, we show the results from the counting of nets stained with Cat-315, PV, and the nets that express both markers.

Figure 4a displays the results for the ipsilateral and contralateral hemispheres. In the ipsilateral hemisphere, the Cat-315^+^ PNN counting showed statistical significance when comparing our four experimental groups (*P* < 0.001) and mainly between stroke STD vs stroke EE (*P* < 0.001). The number of PNNs was reduced in the stroke EE group; however, shams did not present this reduction, suggesting that differential housing conditions “per se” are not responsible for the decrement seen in the stroke EE group. Cell counting revealed a 61% reduction of PNNs in the stroke EE compared to shams. When analyzing for differences in PV^+^ cells, we did not observe any statistical significance. When looking at the numbers of nets positive for both markers, we found statistical significance (*P* < 0.01) and especially when comparing stroke STD vs stroke EE groups (*P* < 0.01). The stroke EE group had 50% fewer PNNs compared to shams and stroke STD groups. In the contralateral hemisphere, no significant difference was observed between the experimental groups and relative to the different marker expressions.

A representative double immunohistochemical staining in Fig. 4b reveals that most of the PV^+^ cells (described in green) are surrounded by Cat-315^+^ PNNs (depicted in red) in a representative rat from the sham STD group. Figure 4c–c″ displays these details in higher magnification, and this coexpression is also seen in the rest of the experimental groups (data not shown).

Overall, these results reveal that the number of PV^+^ cells in the somatosensory regions is neither altered by stroke nor by our EE conditions. Therefore, the reduction in the number of PNNs enwrapping PV^+^ cells that we observe after stroke and EE conditions cannot be attributed to a relative reduction in the number of PV^+^ cells.

### MMP-9 and tPA Proteolytic Activities Are Increased After PT and EE Conditions

**Fig. 5:**
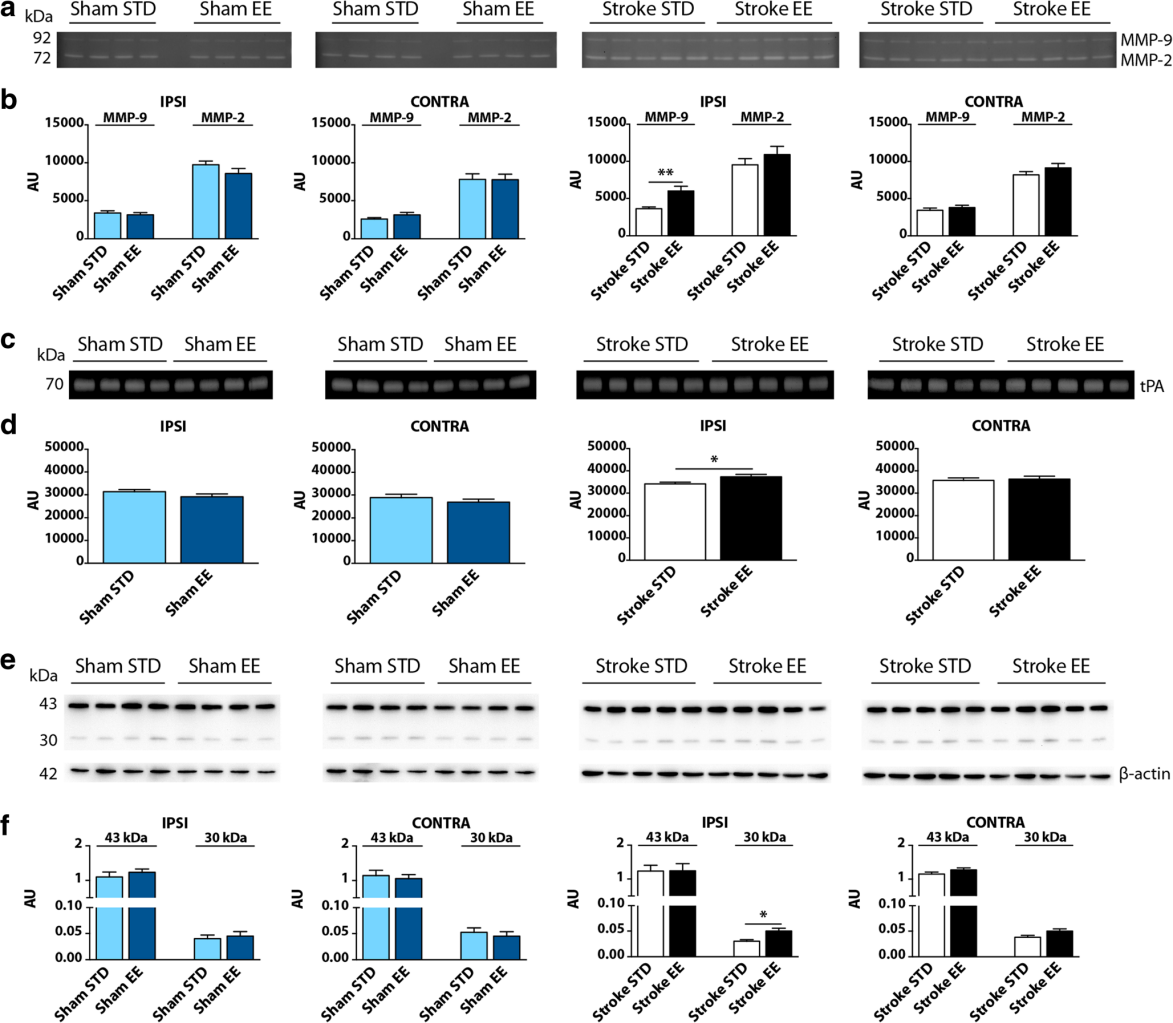
MMP-2, MMP-9, and tPA proteolytic activities in the rat somatosensory cortex. **a** Representative FITC-gel zymography of MMP-2 and MMP-9 appear as two clear bands on a dark background at 72 and 92 kDa (MMP-2 and MMP-9, respectively). **b** Densitometric analyses of MMP-2 and MMP-9 activities in the ipsilateral (IPSI) and contralateral (CONTRA) hemispheres. Significant differences were found in MMP-9 activity in the IPSI hemisphere of stroke injured rats: stroke STD vs stroke EE (MMP-9, **P < 0.01). **c** Representative casein-plasminogen zymography of tPA appear as one clear band on a dark background at 70 kDa. **d** Densitometric analyses of tPA activity in the IPSI and CONTRA hemispheres. Significant differences were found in tPA activity in the IPSI hemisphere of stroke injured rats: stroke STD vs stroke EE (tPA, *P < 0.05). **e** Representative western blot of β-dystroglycan, a substrate of MMP-9, appear as two dark bands on a clear background at 43 and 30 kDa. The 43-kDa band represent the full-length form of the protein and the 30-kDa band represent a proteolytic fragment. **f** Densitometric analyses of β-dystroglycan protein expression in the ipsilateral (IPSI) and contralateral (CONTRA) hemispheres where data were normalized to β-actin expression. IPSI: stroke STD vs stroke EE (30-kDa form, *P < 0.05). Two-tailed unpaired Student’s *t* test, *n* = 4 for sham STD and sham EE, *n* = 5 for stroke STD and stroke EE. *AU* arbitrary units, *STD* standard, *EE* enriched

To examine the influence of EE on the proteolytic activity of ECM proteases such as endogenous gelatinases (MMP-2 and MMP-9) and tPA, gel-zymography was performed in samples from the somatosensory cortex of both ipsilateral and contralateral hemispheres (Fig. 5).

MMPs gelatinolytic activity produced two bands at ∼72 and ∼92 kDa, respectively (Fig. 5a). Since the proforms and active forms of MMP-2 and MMP-9 were not distinguishable on the zymogram, the two bands were quantified as total MMP-2 and MMP-9 activity, respectively. As shown in Fig. 5b, EE led to an increase in MMP-9 activity in stroke EE animals compared to stroke STD animals in the ipsilateral hemisphere (*P* < 0.01, 40% increase).

tPA casein-plasminogen zymography produced one clear band on a dark background at ∼70 kDa which we quantified as total tPA activity (Fig. 5c). As seen in Fig. 5d, tPA proteolytic activity was increased in the ipsilateral hemisphere of stroke EE animals, when compared to stroke STD animals (*P* < 0.05, 12% increase).

These results demonstrate that exposure to an EE for 5 days after PT elevates the activity of the ECM proteases MMP-9 and tPA in the ipsilateral somatosensory cortex but has no effect on MMP-2 activity.

Since the activity of MMP-9 was increased by EE, we next studied the protein levels of β-dystroglycan, a candidate physiological brain target molecule of MMP-9. Figure 5e shows representative western blots of β-dystroglycan in the somatosensory cortex of both ipsilateral and contralateral hemispheres. The β-dystroglycan antibody detected two bands at ∼43 and ∼30 kDa, respectively. Statistical analyses (Fig. 5f) showed no differences in the levels of the 43-kDa form between both shams and stroke groups, in the ipsilateral and contralateral hemispheres, respectively. The 30-kDa form of β-dystroglycan was increased in the stroke EE group compared to the stroke STD group in the ipsilateral hemisphere (*P* < 0.05, 40% increase). These data indicate that PT + EE conditions induce an increased cleavage of β-dystroglycan, a target of MMP-9 activity.

### RT-qPCR Shows Modulation of ECM Protease and Protease Inhibitor mRNA Expressions in the Somatosensory Cortex After PT

**Fig. 6:**
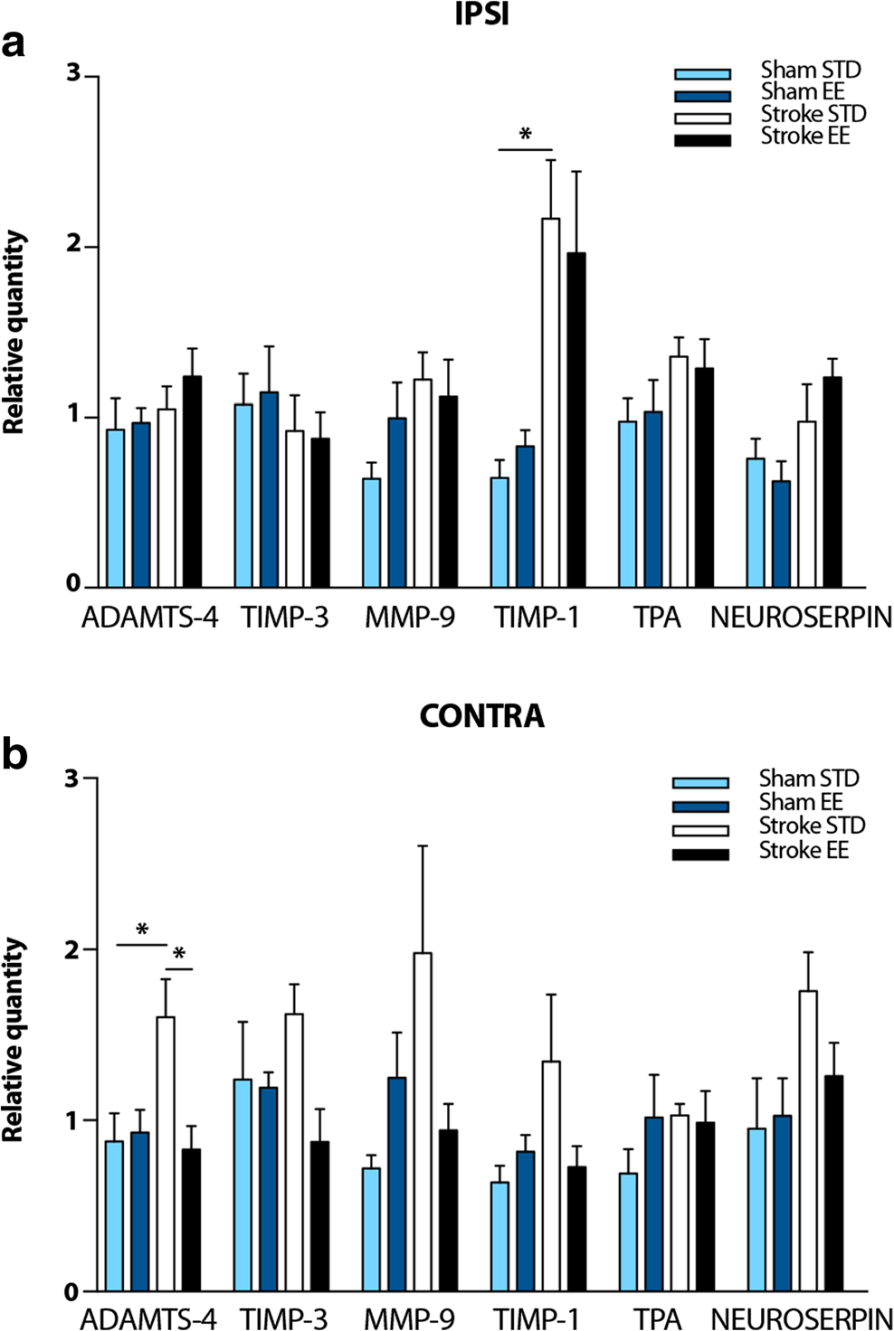
mRNA expression of ECM proteases and protease inhibitors in the rat somatosensory cortex. RT-qPCR of *Adamts4*, *Mmp9*, *Tpa*, and their respective inhibitors *Timp3*, *Timp1*, *Neuroserpin* in the ipsilateral (IPSI) and contralateral (CONTRA) hemispheres. Data from the four experimental groups were analyzed relative to each protease or inhibitor and normalized to the housekeeping genes *Ppib* and *Rpl13a*. **a** IPSI: *Timp1*, sham STD vs stroke STD *P < 0.05. **b** CONTRA: *Adamts4*, sham STD vs stroke STD *P < 0.05, stroke STD vs stroke EE *P < 0.05. One-way ANOVA and Bonferroni’s multiple comparisons test, *n* = 4 for sham STD and sham EE, *n* = 6 for stroke STD and stroke EE. *STD* standard, *EE* enriched

In order to discern if the increased activity of MMP-9 and tPA that we see in the stroke EE group was due to changes in expression of respective ECM proteases, mRNA levels of *Mmp9*, as well as *Adamts4*, *Tpa*, and their inhibitors (respectively, *Timp1*, *Timp3*, and *Neuroserpin*) were studied by RT-qPCR (Fig. 6). Data from the four experimental groups were analyzed relative to each protease or inhibitor of interest. In both hemispheres, mRNA for all our ECM proteases and inhibitors of interest were constitutively expressed in sham and stroke tissue. Figure 6a shows mRNA levels in the ipsilateral hemisphere, where *Timp1* mRNA expression after PT increased significantly by 70% (*P* < 0.05 among our four experimental groups; sham STD vs stroke STD, *P* < 0.05). Figure 6b shows mRNA expression in the contralateral hemisphere. Here, *Adamts4* mRNA expression was statistically 45% higher in the stroke STD group compared to the sham STD group (*P* < 0.05 among our four experimental groups; sham STD vs stroke STD, *P* < 0.05).

In summary, PT induces an increase in the mRNA expression of the ECM protease inhibitor *Timp1* in the ipsilateral hemisphere. In the contralateral hemisphere, PT induces an increase in *Adamts4* mRNA expression which is then downregulated by PT + EE conditions. These data suggest a modulation of the expression levels of ECM proteases and protease inhibitors after PT that could potentially influence the ECM proteases activity and the degradation/reduction of PNNs seen after stroke.

### Stroke Affects the Structure of PNNs and the Activity of MMP-2, MMP-9, and tPA in the Human Brain

**Fig. 7:**
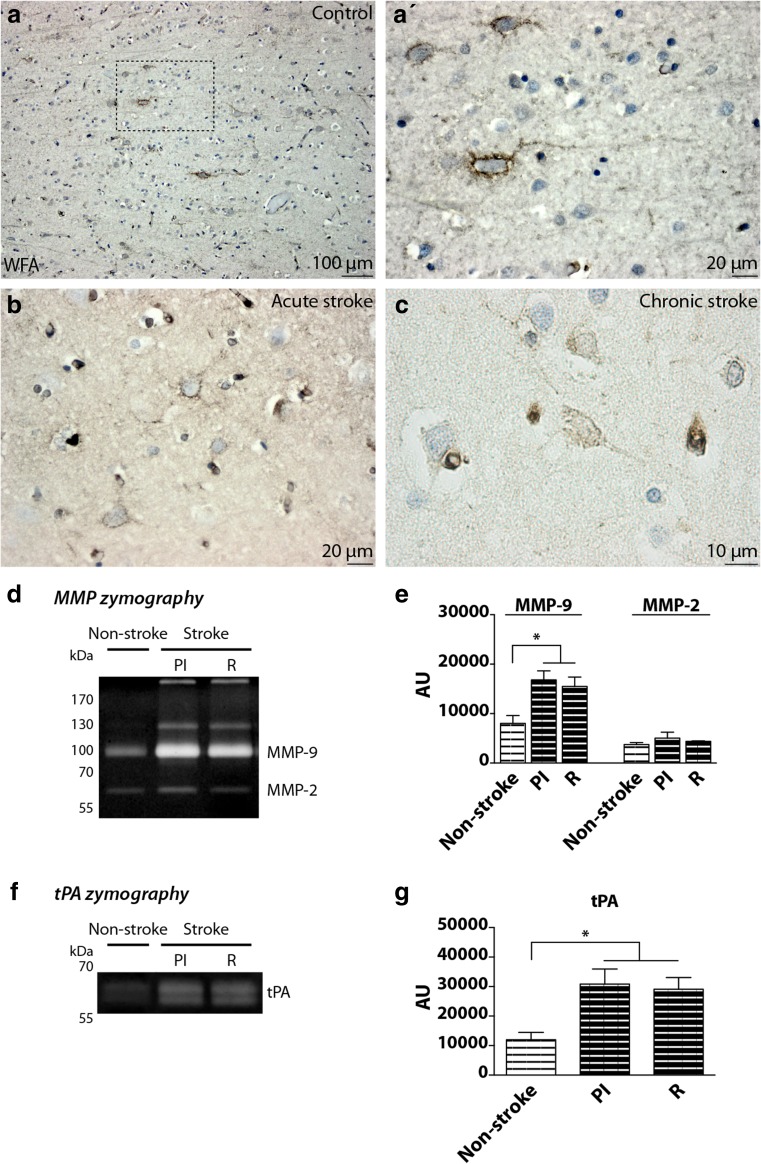
PNN expression and ECM protease activities in the human brain after stroke. Human brains of stroke and non-stroke cases have been analyzed for the expression of WFA, a traditional marker for PNNs and subjected to MMP-2, MMP-9, and tPA gel zymography.** a** Representative picture of a cortical brain section from a control case. WFA^+^ PNNs are visible and dispersed in the cortex of the patient.** a'** Higher magnification. PNNs appear thick, enwrapping the cell-soma and a long dendrite.** b** Representative peri-infarct cortical brain section of a patient who suffered from an acute stroke. PNNs appear thin and disrupted.** c** Representative peri-infarct cortical brain section of a patient after a chronic stroke, where PNNs are barely visible.** d** FITC-gel zymography showing MMP-2 and MMP-9 activity in the human cortex in a control case and in a stroke patient. In the control case, two bands are visible around 100 and 70 kDa. After stroke, two additional bands are visible in the gel (at >170 kDa and at 130 kDa) in the peri-infarct (PI) and in a cortical region remote from the infarct (R).** e** Quantification of MMP zymography (*P < 0.05 non-stroke vs PI or R).** f** Casein-plasminogen zymography showing tPA activity in the human cortex in a control case and in a stroke patient. tPA activity in the control case appears as a faint two-band zymogram between 70 and 55 kDa. After stroke, these bands appear more prominent as seen in both the PI and R regions.** g** Quantification of tPA zymography (*P < 0.05 non-stroke vs PI or R). One-way ANOVA and Bonferroni’s multiple comparisons test, *n* = 3 for non-stroke patients and *n* = 3 for stroke patients

To translate and assess the relevance of our experimental studies to stroke patients (Fig. 7), human cortical brain tissue of stroke and non-stroke patients was analyzed for the presence of WFA, a marker for PNNs in the brain cortex and analyzed with respect to MMP-2, MMP-9, and tPA activities by gel zymography. Figure 7a shows a representative low-magnification micrograph cortical brain section of a control case. In this picture, some WFA^+^ PNNs are visible and dispersed in the cortical tissue of the patient. At higher magnification (Fig. 7a′), the detailed structure of the PNN appears thick, covering the cell-soma and a long dendrite. Figure 7b shows a representative peri-infarct cortical brain section of a patient who suffered from an acute stroke (a few days before death). Here, the PNNs appear thin and interrupted, barely visible, and a similar structure is visible in Fig. 7c which shows a representative peri-infarct cortical brain section of a patient after a chronic stroke (months before death). Figure 7d shows the activity of MMP-2 and MMP-9 in a control case and after stroke of the human cortex. In the control case, two bands are visible around 100 and 70 kDa, in similar positions to our rat study. After stroke, the activity of the two MMPs appears upregulated in the human cortex. Two more bands are visible in the gel (at >170 and at 130 kDa) showing the activity of the proteases in the peri-infarct (PI) and a cortical region remote from the infarct (R). Quantification of the MMP-2 and MMP-9 bands shows increased activity of MMP-9 in stroke patients compared to non-stroke (Fig. 7e; *P* < 0.05 in non-stroke vs PI and non-stroke vs R). tPA activity also appear increased after stroke, in both PI and R regions of the cortex (Fig. 7f). In this case, two bands are visible between 70 and 55 kDa, at the same molecular weight of a tPA control produced by recombinant DNA technology (Actilyse, Boehringer Ingelheim, Germany; data not shown). Quantification of the tPA bands shows increased activity of the protease in stroke patients compared to non-stroke (Fig. 7g; *P* < 0.05 in non-stroke vs PI and non-stroke vs R).

## Discussion

In the present study, we demonstrate that multisensory stimulation, provided by housing rats in an EE for 5 days after experimental stroke enhances recovery of paw placement ability, without affecting infarct size. This is accompanied by increased activity of the ECM proteases MMP-9 and tPA. Concomitantly, protein substrates of these enzymes are modified as evidenced by a decreased number of PNNs enwrapping PV/GABA-containing neurons in the somatosensory cortex, by an increased degradation of aggrecan, the main constituent of PNN, and an increased cleavage of β-dystroglycan. In the following, we will discuss the implication of our results for stroke recovery and rehabilitation of stroke patients.

### Multisensory Stimulation of the Brain After Stroke Enhances Recovery of Neurological Functions

Studies in stroke patients and in animal stroke models show that most recovery from impairment occurs in the first weeks after stroke and that multiple mechanisms are involved in the recovery process [6]. Among others, a sensitive period of postischemic plasticity defined by unique genetic, molecular, physiological, and structural events results in both spontaneous tissue reorganization and increased responsiveness to sensori stimulation and training [46]. In this study, experimental stroke was induced in the forepaw and hindpaw primary motor areas and this lesion caused a complete deficit in the tactile/proprioceptive response to sensory stimuli in rats [12, 36]. EE initiated 2 days after stroke and continued for 5 days, reversed this deficit in the forepaw and augmented the recovery of hindpaw function. Importantly, analysis of the infarct volume among the stroke groups did not show disparities, suggesting that the recovery-promoting effect of EE cannot be attributed to a smaller infarct volume but mainly to mechanisms of brain plasticity, such as experience-driven re-learning [6, 47]. This is in agreement with previous studies referring to environmental enrichment as a stimulating environment with respect to novelty, variety, social interactions, and reward which enhance spontaneous biological recovery in rodents even in the absence of specific training [46, 48–52].

### Multisensory Stimulation of the Brain After Stroke Reduces the Number of Aggrecan^+^ PNNs and Increases Aggrecan Proteolysis

One current hypothesis on impairment of recovery after stroke proposes that an imbalance between excitation and inhibition develops during stroke recovery and leads to a net increase in inhibition of cortical microcircuits of the injured hemisphere [53]. This inhibition is gated by PV/GABA interneurons, frequently enwrapped by PNNs. PNNs carry strong cation-binding properties of the CSPG chains which modulate the activity of PV/GABA cells, and therefore the excitability of cortical microcircuits. PV/GABA cells are also implicated in our study.

CSPGs and aggrecan, in particular, are the main components of PNNs. The structure and number of PNNs are modulated in areas distant from the infarct [12, 54]. CSPGs are also released during reactive astrocytosis after stroke leading to a substantial increase of their expression in the scar tissue which is encapsulating the infarct [15]. The number of PNNs and aggrecan expression increases during synaptogenesis which coincides with the closing of the developmental critical period, a time of enhanced structural and functional synaptic plasticity [55, 56]. Evidences suggest that the poststroke brain is in a state of heightened sensitivity to behavioral experience reminiscent of the critical period [48, 57]. This is strongly supported by our study, where a robust sensori-motor stimulation provided by EE after stroke induced a reduction in the number of Cat-315^+^ PNNs in the somatosensory cortex ipsilateral to the lesion, where the Cat-315 antibody recognizes a carbohydrate epitope carried on the aggrecan core protein [12, 42–45].

Generally, aggrecan that has undergone enzymatic digestion of chondroitin sulfate chains with ChABC is detected with a marked band at >250 kDa, but often multiple weaker bands at lower molecular weights can also be identified on western blots [58]. Here, we demonstrate that aggrecan, a key component of PNNs, undergoes increased digestion following stroke and EE conditions even in cortical regions remote from the lesion. Because aggrecan can be cleaved at multiple sites, several fragments were identified on the western blots from shams and stroke animals. The increased digestion suggests aggrecan proteolysis as an early step in PNNs loss (visible here 7 days after stroke and after 5 days of EE), similarly to what has been described after status epilepticus [45].

Recently, the effects of manipulating CSPGs using ChABC have been investigated after stroke in vivo [59]. It was demonstrated that ChABC treatment was able to promote forepaw sensorimotor recovery and degrade PNNs which may reactivate plasticity. These findings are in line with our results, where environmental stimulation drives functional recovery and plasticity through the degradation of PNNs, which are mainly enwrapping PV/GABA-containing neurons. PV/GABA cells are key regulators of synchronization of neuronal activity in cerebral microcircuits. These microcircuits, gated by PV/GABA interneurons, play an important role in processing information for many tasks such as motor control, cognition, and sensory perception [60], of importance in functional recovery after stroke [61, 62]. Here, we show that under EE conditions, PV-containing neurons were enwrapped by fewer PNNs. The remaining PNNs had poor structural integrity, suggesting that a digestion process may be responsible for the loss of PNNs. Finally, we demonstrated the presence of PNNs in the human brain, which appear similar to the ones seen in our experimental model. In control patients, these nets were thick and enwrapping the entire cell soma and proximal dendrites, as reported earlier [63]. After stroke, the nets appeared thin and disrupted, as seen in the rat brains after experimental stroke. The degradation of PNNs could change the kinetics and fast-spiking characteristics of the PV/GABA cells [60]. Together, these data strongly suggest that multisensory stimulation provided by EE induces a degradation of aggrecan-containing PNNs, opening-up a critical period for plasticity after stroke in the adult brain.

### Multisensory Stimulation of the Brain After Stroke Activates ECM Proteases

ECM proteases and their inhibitors are involved in PNN modulation after stroke. PNNs can be envisaged as targets for pharmacological interventions as observed in seizure genesis [64]. Different proteases have been proposed to contribute to ECM modulation and degradation after brain injury, among others tPA, ADAMTS-4, MMP-2, and MMP-9 [25, 28–31]. In our study, the activity of tPA and MMP-9 is increased in the ipsilateral somatosensory cortex of the stroke EE group compared to the stroke STD group. These proteases might then be involved in the remodelling of ECM structures and changes in the integrity of PNNs that we observe after stroke and EE conditions in regions remote from the infarct. We also showed an increased activity of tPA and MMPs after stroke in human samples, suggesting similar processes in the human brain following stroke. tPA expression and activity in the healthy human cortex have been reported earlier [65] and MMP-2 and MMP-9 activities have been studied in the ischemic core of stroke patients [66]. Our results in stroke patients highlight a remarkable presence of tPA, MMP-2, and MMP-9 activities in peri-infarct zones and most importantly in regions remote from the infarct. This suggests a predominant remodelling of the abovementioned areas after stroke in patients which persists for months after the event.

Despite data implicating MMP-9 in neuronal/synaptic plasticity, not many synaptic targets for its enzymatic activity have yet been identified. However, recent studies have suggested that this enzyme may digest the full-lenght β-dystroglycan to release a 30-kDa product, mainly expressed in the postsynaptic membrane and in close proximity to MMP-9 [32]. β-Dystroglycan is a cell surface protein protruding from postsynaptic membrane and bound (probably throughout the synaptic cleft) to presynaptic neurexins, implicated in neurotransmitter release and long-term potentiation (LTP) [67]. In this study, we observed increased levels of the 30-kDa form of β-dystroglycan, which may have been generated by MMP-9, similar to previous reports where enhanced neuronal activity was challenged [32, 68]. One explanation of this result could be that the multisensory stimulation provided by EE induced a MMP-9 activation-dependent cleavage of the full-length form of β-dystroglycan in the ipsilateral hemisphere. A fine structural immuno-colocalization of β-dystroglycan and MMP-9 has been previously shown by electron microscopy in the rat brain [32]. In addition, β-dystroglycan cleavage is abolished after a MMP-9 inhibitor treatment [32] and in MMP-2 and MMP-9 knockout mice [69], further suggesting a role of MMP-9 in β-dystroglycan cleavage.

MMP activity is tightly controlled by endogenous tissue inhibitors of MMPs (TIMPs), a family of proteins comprised of four members (TIMP-1 to TIMP-4), which inhibit multiple MMPs and display different interactions and substrate specificity with MMP proenzymes [70]. tPA activity in the CNS is regulated by neuroserpin, a member of the serine protease inhibitor (serpin) family that is secreted from the growth cones of neurons [71]. It is important to note that protease expression is not always indicative of net proteolytic activity, since the latter is an outcome of expression levels, posttranslational activation, and protease inhibition [34]. Interestingly, mRNA expression analysis showed an upregulation of *Timp1* in the ipsilateral hemisphere after stroke. *Timp1* has been shown to be upregulated following focal ischemia, a process that could be part of a general neuronal response that mediates tissue reorganization [72]. In our study, the upregulation of *Timp1* supports this hypothesis and suggests that the observed changes in MMP-9 and tPA activities are regulated by the expression of the corresponding inhibitors rather than by translation of ECM protease genes. In the contralateral hemisphere, *Adamts4* mRNA expression was upregulated after stroke, but not after stroke and EE conditions, a tendency also seen in *Timp3* and *Neuroserpin*. This suggests a transcallosal influence of the injured hemisphere on the contralateral hemisphere protease mRNA expressions, which is prevented by multisensory stimulation exerted through an EE. *Adamts4* mRNA expression is upregulated in astrocytes following transient middle cerebral artery occlusion in the rat [73], but this is the first study reporting their modulation in regions remote from the lesion after permanent stroke. Similarly, *Timp3* and *Neuroserpin* are upregulated in penumbral cortical neurons following stroke in the ipsilateral hemisphere [74, 75].

## Conclusions

In conclusion, this study reports that experience-dependent plasticity induced by housing rats in an EE after stroke reduces the number and structure of aggrecan-containing PNNs surrounding PV/GABA neurons in areas remote from the ischemic lesion. This reduction is accompanied by an upregulation in the activity of ECM proteases such as tPA and MMP-9 and a modulation in the expression of ECM proteases and inhibitors such as *Adamts4* and *Timp1*. Similar results were seen in brain tissue from stroke patients. We conclude that multisensory stimulation of the brain after stroke, which provides enhanced recovery of lost function, involves the modulation of ECM proteases and protease inhibitors. Elucidating the mechanisms of ECM remodelling after stroke may contribute to the development of new therapies supporting rehabilitation of stroke patients.

## Electronic Supplementary Material

ESM 1 (DOCX 102 kb)
