# Supplementary material for: Extracellular Matrix Modulation Is Driven by Experience-Dependent Plasticity During Stroke Recovery
Source: Mol Neurobiol. 2017 Mar 13;55(3):2196–213. doi: 10.1007/s12035-017-0461-2 (PMC5840227; doi:10.1007/s12035-017-0461-2)

**Extracellular matrix modulation is driven by experience-dependent plasticity during stroke recovery**

Miriana Jlenia Quattromani^*^, Mathilde Pruvost, Carla Guerreiro, Fredrik Backlund, Elisabet Englund, Anders Aspberg, Tomasz Jaworski, Jakob Hakon, Karsten Ruscher, Leszek Kaczmarek, Denis Vivien, Tadeusz Wieloch

^*^Corresponding author

Affiliation: Laboratory for Experimental Brain Research, Division of Neurosurgery, Department of Clinical Sciences, Lund University, BMC A13, 22184 Lund, Sweden

Email: miriana.quattromani@med.lu.se

**Supplement Table 1**

Rat study. Physiological parameters of group I and II. Data are presented as mean ± SEM.

| **Parameters** | **Time-point** | **Sham STD** | **Sham EE** | **Stroke STD** | **Stroke EE** |
| --- | --- | --- | --- | --- | --- |
| *Temperature (°C)* | End of PT | 37.4 ± 0.1 | 37.6 ± 0.1 | 37.6 ± 0.1 | 37.6 ± 0.1 |
| *Body-weight (g)* | Pre PT  Day 2 | 397 ± 8  414 ± 7 | 411 ± 9  420 ± 9 | 404 ± 7  396 ± 10 | 363 ± 12  360 ± 12 |
|  | Day 7 | 412 ± 9 | 431 ± 25 | 423 ± 6 | 378 ± 11 |

**Supplement Table 2**

Human study. Physiological parameters of stroke patients

| **ID** | **Age** | **Patient** | **Matter** | **Brain sample** | **Hemisphere** | **Cause of death** | **PM delay** |
| --- | --- | --- | --- | --- | --- | --- | --- |
| *1* | 65 | Non-stroke | Grey and white | Cortex | - | AMI and renal failure | 1d |
| *2*  *3*  *4* | 80  83  74 | Non-stroke  Stroke  Stroke | Grey and white  Grey and white  Grey and white | Cortex  Cortex  Cortex | -  Ipsi  Ipsi | AMI  AMI and urothelial carcinoma  AMI and pan pneumonia | 3d  3d  10d |
| 5  6  7  8  9  10 | 78  80  81  73  61  56 | Stroke  Non-stroke  Non-stroke  Stroke  Stroke  Non-stroke | White  White  Grey and white  Grey and white  Grey and white  Grey and white | Cortex  Cortex  Cortex  Cortex  Cortex  Cortex | Ipsi  -  -  Ipsi  Ipsi  - | AMI and heart failure  AMI and heart failure  Circulatory arrest  AMI  Brain-stem hemorrhage  AMI | 2d  2d  2d  5d  3d  1d |

**Supplement Fig. 1**

Determination of the most appropriate reference genes in our different groups using the geNorm M algorithm (qbase+ analysis) on 10 samples and 8 reference targets. **a** The optimal number of reference genes in our experiment is 2 as the pairwise variation V value for V2/3 is under 0,15. **b** The best housekeeping genes to study stroke in two different housing conditions are Rpl13a and pPib, the geNorm M-value is under 0.5 for these genes

**a** **b**


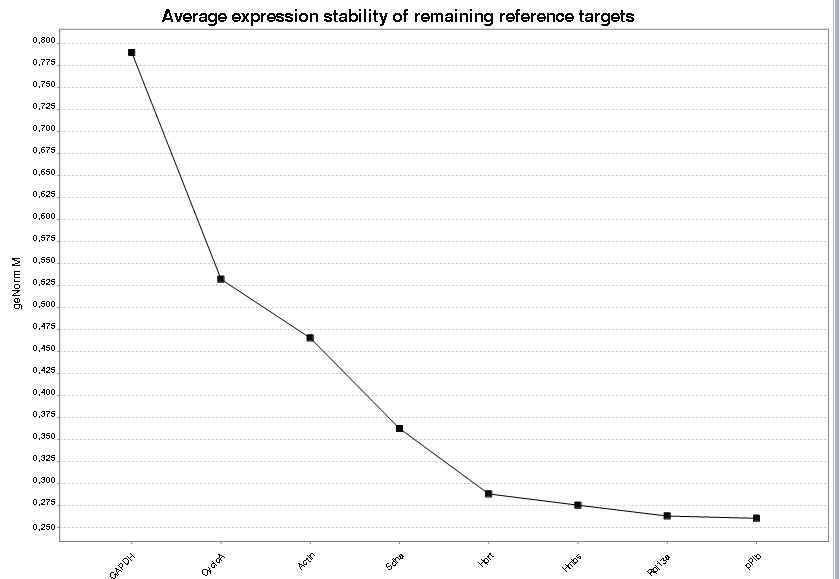

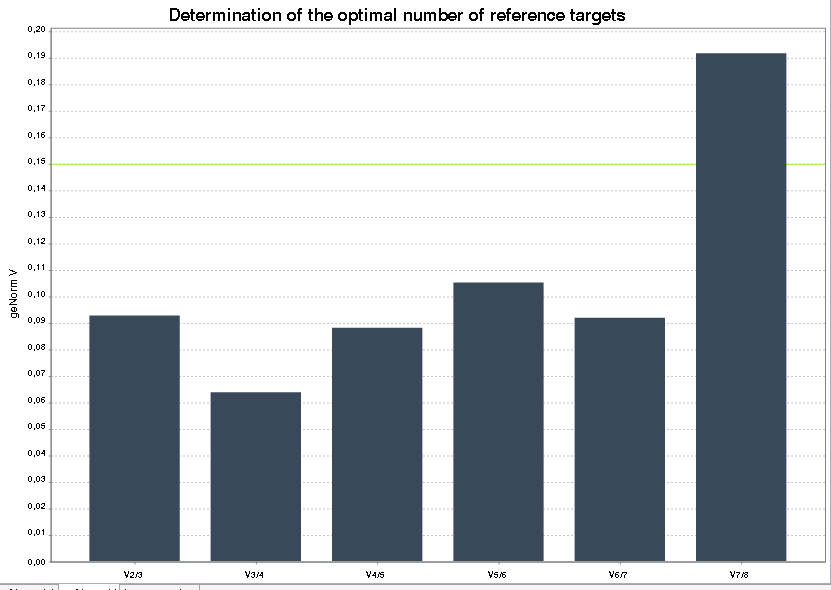

Supplement: Supplementary file 1 — (DOCX 102 kb) [file 12035_2017_461_MOESM1_ESM.docx]
